# Developing a quality and safety surveillance system: A protocol for a realist review and synthesis of the literature

**DOI:** 10.1371/journal.pone.0321720

**Published:** 2025-05-29

**Authors:** Busra Ertugrul, Jaspreet Kaur Dullat, Therese McDonnell, Marcella O’Dowd, Eilish McAuliffe

**Affiliations:** 1 UCD IRIS Centre, School of Nursing, Midwifery & Health Systems, Belfield, Dublin 4, Ireland; 2 National Quality and Patient Safety, Health Service Executive, Dublin 8, Ireland; AJUMS: Ahvaz Jondishapour University of Medical Sciences, IRAN, ISLAMIC REPUBLIC OF

## Abstract

**Background:**

Patient harm as a result of unsafe care remains a growing global health concern, and traditional efforts prioritising tracking errors and voluntary reporting to detect adverse events result in very low error detection rates. In attempting to address this, some countries have developed patient safety surveillance systems where several data sources are combined and interrogated to detect errors and deterioration as well as to highlight good practices. The development of such systems requires overcoming various technological, executive, financial, political, and behavioral challenges. The process of developing quality and safety surveillance systems is an important one that is worthy of research that enables the sharing of learning with countries embarking on the development of such a system.

**Aim:**

This is the protocol of the realist review that aims to develop a programme theory that explores the underlying mechanisms and processes for developing a quality and safety surveillance system in healthcare settings.

**Design:**

This paper presents the research protocol for the quality and safety surveillance system development. The realist synthesis method will be used to synthesize the evidence in the literature.

**Methods:**

Initial programme theory will be developed based on a literature review, stakeholder consultations with the programme designers and developers, project meetings, the theory of change model that underpins the programme, and an understanding of systems theory to identify the context and mechanisms that cause quality and safety surveillance systems to succeed or fail in general healthcare settings. Three-phase iterative searches of PubMed, PsycInfo, Central, CINAHL, and Grey literature will be conducted. The studies in any setting and for any patient group will be included. The snowball technique will also be used to search the reference list of the related studies. The search period ranges from 1 January 2000 to 31 December 2023. Results will be reported following RAMESES and PRISMA‐ScR guidelines.

## Introduction

Patient harm because of unsafe care remains a growing global health concern, and it is one of the top causes of death and disability worldwide [1]. Twenty years after the report “To Err Is Human”, despite multiple initiatives to improve patient safety, one out of every ten patients experience an adverse event while receiving hospital care in high-income countries. Adverse or transient harm events in the US affect about 25% of all Medicare patients [2]. Establishing an effective method for national patient safety measurement has become increasingly crucial as interest in improving patient safety has grown [3]. While there are many differences between care systems worldwide, improved data in systematic monitoring and assessment of adverse events, consistent screening for risk factors, and outcome measurement are among the emerging key characteristics [2].

Surveillance processes involve collecting data, data quality evaluation, managing data, data assessment, the interpretation of analysis results, dissemination of information, and the implementation of the data for health initiatives [4]. Countries such as Canada, the U.S., and the U.K. have used surveillance systems in population health care and reported the surveillance system findings collected from several European countries [5–7]. However, it has been stated that national health statistics records are not a reliable source of surveillance, even in high-income countries [6]. Traditional efforts that prioritize tracking errors and voluntary reporting to detect adverse events result in very low levels of error reporting [8–10]. These errors are usually subjected to comprehensive root cause testing several years after they occur, with findings that can be inadequate and inaccurate and solutions that are generally overlooked. The safety reports evidence that assessing patient safety based on prior errors or injuries is inadequate [8]. Without robust data and information systems, little progress would have been accomplished in quality and safety management.

To develop a safety surveillance system, safety management prioritises a better understanding of the components of safe practice that can be quantified and monitored proactively [11]. Health systems should work to guarantee the level of safety of care provided and use the data sets that are already accessible to find patterns in patient safety data that could indicate the level of safety provided in the future [12]. The collection of data is not an endpoint; thus, it is crucial that the monitoring and analysis of data, which will guide the creation of patient safety profiles for cohorts of patients, services, and clinicians, be part of a cycle of quality improvement. Informatics research has grown rapidly to advance surveillance systems in a variety of ways, including facilitating access to new data streams from clinical and other sources, automating surveillance processes such as case detection from free text, and enabling the rapid dissemination of surveillance-generated information to a diverse set of stakeholders [13]. In this respect, WHO encouraged building digital surveillance systems that solve basic difficulties such as standardisation, interoperability, performance, needs assessment, and growth [1]. The present WHO initiative, one of the primary goals of the WHO Global Patient Safety Action Plan 2021–2030, provides a continuous flow of information and knowledge to promote risk mitigation, a reduction in unnecessary harm, and improvements in care safety by developing patient safety surveillance systems.

Patient safety surveillance is described under the WHO’s Strategic Objective 6, “Information, Research, Risk Management”. The main characteristics of the systems described are incident reporting and learning, information, surveillance, research programme, and digital technology focused on patient safety [1]. In this regard, some countries developed patient safety and quality initiatives. Medicare Patient Safety Monitoring System has been developed and improved to provide rates of 21 specific hospital inpatient adverse event measures, with the consistent measurement of the safety of inpatient care, and currently serves as the major national-level patient safety data source in the US [2]. New Zealand developed a health system quality dashboard focused on seven measurement areas on the safety dimension [14]. However, digital surveillance systems in quality and safety are still in the early stages, and little emphasis is given to their development. Ironically, the emergence of numerous software systems, like electronic health records and incident reporting systems, brings in many methods for illustrating patient safety incidents [15]. Although the widespread adoption of electronic systems was anticipated to simplify the reporting process, the absence of clear uniform standards in patient safety reporting systems makes the process difficult [2]. In this sense, WHO does not suggest a pathway to develop patient safety surveillance systems rather, it seeks to establish, synergise, and scale them up. Understanding the user experiences of the systems developed, especially in data use, data validity, quality improvement initiatives, and public reporting, will shed light on the systems to be developed next. Thus, comprehending their underlying mechanisms of operation, how they evolved (e.g., with consumer or clinician involvement), and the situations in which they are employed is important in identifying how safety and quality surveillance systems can be developed in different healthcare settings and patient groups. Thus, our study poses a significant step forward in identifying essential features of developing quality and safety surveillance systems.

Quality improvement during a new system development requires overcoming various technological, executive, financial, political, and standardisation challenges [16]. Medical Research Council guidance emphasises that complex interventions should have a compatible theoretical basis, with theory being used systematically in their development [17]. Complex interventions are characterised by multiple parts interacting with each other and the political, historical, social, and geographic contexts in which they are situated to produce outcomes [18]. Developing quality and safety surveillance systems is a complex, large-scale intervention involving system transformation and organisational culture change within health services. Assessment of the implementation of complex interventions in healthcare is important for implementation planners and policymakers because it provides a theoretical framework for the process. The need to develop safety and quality surveillance systems in healthcare settings is well articulated in the literature [19]. Nevertheless, systematic reviews fail to offer information about how programmes work across various groups and contexts, information that could helpfully inform policy choices. Traditional reviews focus primarily on the evidence for the effectiveness of an intervention and rarely consider situations and settings or the how and why questions [20]. This realist review will delve deeper to explore how and why surveillance systems work or don’t work.

While realist evaluation has been employed to investigate the effects of complex interventions in healthcare, such as programmes for building new systems, realist approaches to design are increasingly reported [21,22]. This review will provide the potential learning from similar programmes’ experiences, successes, and failures to understand the important contexts, mechanisms, and outcomes to be followed in successful intervention and policy design to ensure patient safety systems successfully work at national, regional, and organisation levels. A realist review will be used to develop theoretical causal explanations to be used in the form of recommendations and intervention design for the monitoring and evaluation phase of the Quality and Safety Signal System (QSSS) programme. QSSS is a collaborative project that will help to integrate, analyse and display quality, safety, and operational data from multiple different datasets in one place in an Irish context.

Due to the time consuming nature of full realist reviews of literature and policymakers’ and planners requirements for timely information to inform decisions, the rapid realist review methodology was developed (RRR) [20]. This paper provides a protocol for an RRR that examines what contextual factors and mechanisms are essential for the development and implementation of an effective safety and quality surveillance system. Realist evaluation includes creating, testing, and refining programme theories using several context-mechanism-outcome (CMO) configurations. These configurations indicate how and why the intervention is supposed to work, for whom, and under what circumstances. This is because it is acknowledged that interventions alone have no decisive effect on the outcome. Rather, interventions provide resources to recipients, and outcomes are determined by how the resources are used or not used by the recipients, which changes depending on the context [23].

## Aims

This research aims to develop a theory that explores the underlying mechanisms and processes in developing a quality and safety surveillance system in any healthcare setting. The theory will provide a deeper understanding of the relationship between quality and safety surveillance system interventions, underlying system mechanisms generated by those interventions, and the resultant impact on outcomes in healthcare settings. The review question is “What works for whom in what conditions; why, to what extent, and how in the development, implementation, and use of quality and safety surveillance systems?”

### Specific objectives are

- To explore the key mechanisms influencing or driving successful quality and safety surveillance system implementation in health care settings. (Mechanism)
- To understand under what conditions and to what extent the quality and safety surveillance system works best in any healthcare setting. (Context)
- To explore what are the dominant outcome patterns in the identified contexts. (Outcomes)
- To explore the system developers’ challenges and what they can learn about overcoming them.

## Method

### Design

Realist methodology aids the understanding of complex interventions by asking “how, why, for whom, to what extent, and in what context” complex interventions are successful. Saul et al. (2013) modified and expanded on Pawson’s original methodology as a ‘Rapid Realist Review’ (RRR) tool for policymakers offering prescriptive advice in responding to time-sensitive and emerging issues with limited resources. Because the project is large-scale and has a time limitation, Saul’s tool (focused on identifying group interventions related to outcomes of interest for policymakers) was chosen. Thus, the review will be operated by using the work backward method from the desired outcome to ‘families of interventions’ (I) that can be implemented to produce those outcomes, supported by a theoretical understanding of the contexts (C) within and mechanisms (M). By doing this, the methodology shifts its emphasis from developing theories that can be identified as theory-driven to contextually relevant interventions likely to be linked to specific outcomes within a given set of constraints [20]. RRR recognises the use of multiple evidence sources such as Grey literature or official webpages. The involvement of stakeholders and expert panels in the review process is another key strength of RRR. By enabling stakeholder engagement, RRR recognises a user group voice for theory refinement and reality checks to be pragmatic. With stakeholder engagement, it aims to provide policy-relevant findings at a level of abstraction that can be transferred across settings. The extent of stakeholder engagement throughout the realist research varies based on the programme needs to ensure both research focus and findings are useful [24]. In this study as a first step in the national level system project, we engaged in many phases as suggested by Saul [20]. The realist review methodology and the expected contribution from stakeholders in the following phases were made clear to create clear ways to communicate through the weekly and then biweekly project meetings, as highlighted by Abrams et al. [25]. Stakeholder’s contribution will be sought in (a) defining the scope and concept mining process of the review, (b) assisting with the selection of the relevant keywords, (c) suggesting and sharing relevant literature, (d) interpreting the review findings; (f) disseminating outcomes at a national conference. Stakeholders will not be involved in the selection of articles for inclusion in the review, as extraction and synthesis steps will be undertaken solely by the research team.

The National Quality and Patient Safety Surveillance System development project (the case that will inform our programme theory) has five workstreams ‘Data Governance, System Design, Clinical Advisory Group, Change Management, and Signal Design’. The workstream members were involved in the development of this review protocol to increase the clarity and knowledge of the methodology and outcomes of the review and improve the relevance of the review to the project. QS Signals project Change Management workstream leads were also involved in the protocol development process in determining inclusion and exclusion criteria [20,26,27]. Workstream members will be referred to as expert panel members throughout this protocol.

### Search strategy

The RAMESES protocol will be used as a guide in this study [27]. The process will follow developing a theoretical framework, a search strategy, choosing and evaluating documents, extracting data, analysing and synthesising the evidence, and presenting and sharing revised theory steps. Ethical permission was not necessary for the realist review of the literature. The University College Dublin Research Ethics Committee (ref: LS-LR-23–184-McAuliffe) and the Reference Research Ethics Committee Midlands Area and Corporate (Regional Health Area B) (ref: RRECB1123LH) have provided a favourable ethical opinion for the realist interviews to be conducted for the IPTs testing phase of the project.

### IPT development

**Fig 1:**
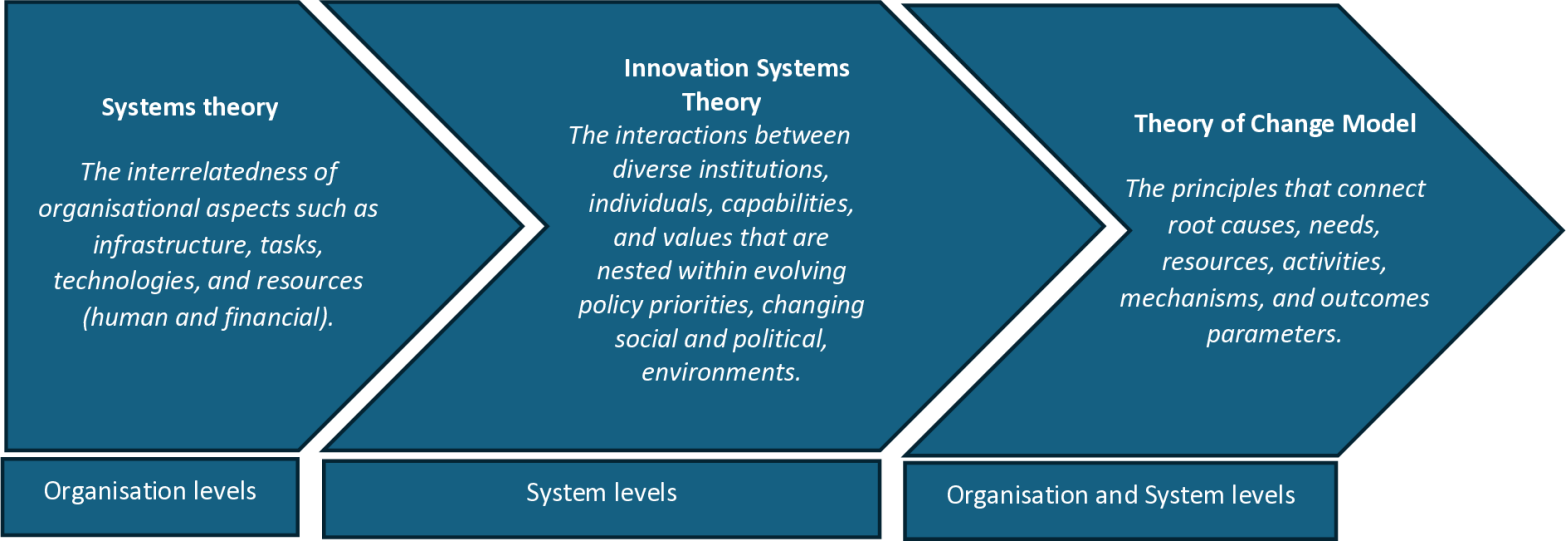
Theoretical framework of the programme.

An intervention is seen as a theory in a realist synthesis since it is predicated on the idea that doing X in this manner will lead to Y. Thus, initial programme theories (IPTs) are presumptions that help explain how the initiatives operate. They serve as the analytical building blocks in realist evaluation, and by connecting configurations (CMO), hypotheses are tested and then enhanced. Initial programme theory will be developed based on a literature review, stakeholder consultations (expert group), project meetings, and the theory of change model that underpinned the National Quality and Patient Safety Surveillance system to identify the context and mechanisms that cause quality and safety surveillance systems to work or fail in general healthcare settings. Theoretical frameworks can be helpful to organise IPTs, understand complex change processes, and improve coherence, quality, and transparency. Pawson and Tilley suggested (1997) that existing theory could and should inform programme hypotheses. The construction of a broad conceptual framework at an early stage can help IPT organise within a hierarchy, understanding complex processes of change, therefore improving coherence, quality, and transparency in realist research, particularly concerned with multifaceted [28,29]. A theory of change model was selected from the programme team because it will help to evaluate the process and clarify the reasoning behind the planned work [30]. In line with the initial literature review and meetings with expert panel members, the research group purposively searched for both the micro and macro-level processes theories to support an understanding of what may work to deliver this kind of implementation. Because this review will inform a national level system, the system theory is chosen to be used to provide national, regional, and organisational level CMOs. Thus, systems theory and innovation systems theory frameworks will be used to guide the development of programme theories by highlighting key concepts and relations that might be influential (Fig 1) [31–34].

### Search strategy

Before the commencement of the review, the research team conducted discussions to decide on the terms to include in the literature search, inclusion and exclusion criteria, and a clear definition of the RRR’s scope. The primary researcher (BE) conducted a preliminary review of the literature to gain an understanding of the different quality and safety-focused surveillance systems. *Concept Mining:* After the non-systematic scoping of the literature on the quality and safety surveillance systems, programme protocols and documentation, and meeting with the key stakeholders, by considering the context, mechanism, and outcomes to build theory, the team developed keywords and refined them in light of the emerging data. Because of the novelty of big data use in health systems and the aim of the programme at the national level, all healthcare settings were included in the search. Less emphasis placed on ‘key words’ to guide search, more focus on identifying context, mechanisms and outcomes to build theory. Inclusion/exclusion criteria refined in light of the emerging data and as programme theories are refined. Articulating the concepts with the questions such as “what the program is?”; “who is the supposed target?”; and “what is the supposed outcome?” was the essential first step in theory development the inclusion and exclusion criterias were developed [35]. The search string included ‘concept’ and ‘context’ as suggested by PRISMA ScR guidelines to search for relevant literature, as the search will not focus on any specific population [36–38]. ***Concept:*** The scoping review will identify mechanisms, conditions, contextual factors, outcomes, and challenges of successfully implementing a safety and/or quality surveillance system. ***Context:*** In line with this, this review will focus on the studies within any healthcare setting (e.g., acute care, aged care, community, mental health, primary care) or for any illness or patient group (Table 1).

**Table 1:** Inclusion and exclusion criteria of the RRR.

*Population*	Will not focus on any specific population.
*Concept*	Surveillance systems on quality and patient safety will be included. Studies that can provide information on the context/mechanism/outcomes regarding the working of Patient Safety and Quality Surveillance Systems. Infectious disease surveillance and population surveillance studies that only focus on measuring mortality or morbidity will be excluded. Population surveillance focusing on post-marketing safety evaluation of drugs, vaccinations, COVID-19, and medical devices will be excluded.
*Context*	Healthcare settings.
*Study type*	Studies in English only after 2000 to the present. Studies focus on the human population. Studies in any methods, editor papers, and Grey literature.
*Search Strategy*	Pubmed: (“Adverse event surveillance system*”[tiab] OR “safety surveillance system*”[tiab] OR “patient safety surveillance”[tiab] OR “quality surveillance system*”[tiab] OR “safety surveillance program*”[tiab] OR “quality surveillance program*”[tiab] OR “safety reporting system*”[tiab] OR “error reporting system*”[tiab] OR “Near Miss Surveillance System*”[tiab] OR “enhanced surveillance system*”[tiab] OR “evaluating surveillance system*”[tiab] OR “Risk Assessment Monitoring System*”[tiab] OR “Patient safety monitoring system*”[tiab] OR “defects surveillance system*”[tiab]) NOT (“Public Health Surveillance”[Mesh] OR “Public Health Surveillance”[tiab]) AND (“Health Facilities”[Mesh] OR “Health Services”[Mesh] OR “Health center*”[tiab] OR “health organisation*”[tiab] OR “health organization*”[tiab] “Primary Health Care”[MeSH] OR “General Practice”[Mesh] OR healthcare[tiab] OR “health care”[tiab]) NOT (“Vaccines”[Mesh] OR “COVID-19”[Mesh] OR “Vaccines”[tiab] OR “COVID-19”[tiab])

#### Initial search.

An exploratory and non-systematic scoping of the literature (informal literature search) and consultation with stakeholder groups have been done to establish the “lay of the land” [26]. The preliminary search focuses on grey literature, which may be difficult to find using a traditional search strategy. The questions “What the programme is?” and “What is the supposed outcome?” will be updated iteratively according to new data. To guide the final search strategy, an initial search will be run in PubMed, CINAHL, CENTRAL, PsycINFO, and SafetyLit databases to discover words and phrases found in the title and abstract of publications that are likely to be included in the review. This step entailed comprehending the subject matter, identifying provisional programme theories, looking at the governmental plans and guidelines, browsing the literature, talking with experts and stakeholders, and developing the concept and vocabulary [23]. An initial search used the following keywords and their synonyms: safety and/or quality surveillance systems and health care settings. This was followed by examining text words found in the titles and abstracts of retrieved articles to find the synonyms of the surveillance system in the literature. After several searches, Embase was excluded as it broadly focuses on drug studies. The search strategy, databases, inclusion and exclusion criteria, and keywords were discussed with the librarian. Search terms and inclusion/exclusion criteria have been iteratively updated as the search proceeds to identify the documents most pertinent to the review topic without omitting many that may be crucial to include.

#### Second search.

The keywords identified will be applied to the second formal search. These searches will be recorded for inclusion in the final PRISMA flow chart. A second search will be conducted across all involved databases, using all relevant recognized keywords and index terms: PubMed, CINAHL, PsycInfo, CENTRAL, and SafetyLit. Outside of peer-reviewed journals, sources of evidence will be significant in this search since, due to the nature of the topic, relevant frameworks, guidelines, and explanatory papers may not be published in peer-reviewed journals. WOS, Grey Literature Report, Google Scholar, OpenGrey.eu or Greylit.Org., and organisational webpages (e.g., Annual Reports of WHO, AHRQ, NHS, PSNet) search will also be conducted. Integrating the SafetyLit database guarantees the capture of publications specifically linked to safety that are not otherwise collected. The articles written in English from 2000 to the present will be included to focus on quality and safety surveillance system development and/or implementation in healthcare following the publication of To Err is Human [39]. Quantitative and qualitative evidence will be included, and a snowball approach will be used in which one reference leads to others. If the manuscript is unavailable, the authors will contact to seek sources. *Reference Search:* The reference lists of all recognised papers will be searched for additional investigations.

### Selection and appraisal of documents

Because the realist review does not strictly adhere to the inclusion criteria, search terms and inclusion/exclusion criteria will be iteratively modified as the search progresses to identify the documents most relevant to the review topic while excluding many that might be important to include. Any part of the data will be evaluated for its contribution based on two factors: relevancy and rigor. Relevance refers to whether the study contributes to the development and/or testing of theories, while rigor refers to the reliability and validity of the data generation process. In line with Pawson’s suggestions, the criteria for inclusion will be: is the evidence in this implementation “good enough and relevant” for inclusion? Discrepancies about the relevance of manuscripts will be resolved through discussion in the review group. It is challenging to first reduce the number of papers due to the diversity of the sources and the realist review’s emphasis on relevance and rigor. Thus, the framework will be piloted with the first 20% results in screening [26]. This could happen while the data are being examined. Thus, the analysis stage will follow the selection and evaluation stage.

The researchers (BE, JKD) will review the title and abstract before deciding on a full-text retrieval. After analysing the complete text, the team will assess if the evidence presented is sufficient to be included in the review in the second screening. Documents retrieved via grey literature searches without an abstract will be subjected to full-text examination. Screening titles and abstracts may need to include important causal insights or theoretical references. Thus, no paper is truly discarded; they are held in ‘reserve’ if they can contribute later [26]. Studies obtained from the databases will be uploaded to the covidence review software. The review questions and framework will be followed by the review team in Covidence platform to provide transparency in the selection of the resources. They will be screened according to inclusion and exclusion criteria in this database. All studies in the iterative search process will also be collected and managed in the EndNote library. The importance of the expert panel’s and reference group’s help in the search process cannot be underestimated with respect to conducting a rapid review, so rapid feedback from both reference group and expert panel members is necessary for conducting rapid realist reviews.

### Data extraction process

Before data extraction, the following processes will be performed: The first step is to create a standardised extraction form. Second, pilot tests the form with two or more reviewers on two to three manuscripts to ensure consistency. This step is ‘digging through’ the data using knowledge to find essential concepts, words, and introductory theories that provide background information on the topic of interest. The extracted data will explain how and why the programme may have operated under a given concept. The extraction template will include programme impacts, implementation procedures, particular programme context elements, and how these contexts shape the mechanisms that might drive change.

After the initial round of extractions, the review team will outline significant themes and findings, focusing on contextual factors, mechanisms, and interactions that affect outcomes. As documents are extracted, forward and backward citation searches on important articles will be carried out. Data extraction is not a linear process and may be performed several times before being synthesised [26]. Two researchers will evaluate the full text independently against the inclusion criteria. When there are disagreements between two reviewers, a consensus meeting will be held with the expert group to assess the source and determine its eligibility. The completed data extraction table of a programme theory will be included as an appendix in the publication of the review. The Protocol of this scoping review was developed in line with the best-practice guidelines [36–38]. The PRISMA ScR Checklist is used as a guide to explain review methodology [40].

### Analysis and synthesis processes

A realist data analysis aims to examine data through the lens of realism ideas. Therefore the analysis will find repeated patterns in CMO configurations within or across the review data: What resulted (outcome) from the various programmes or processes regarding contexts and mechanisms? What method or reason led to these results (mechanism)? What settings (contexts) for development and intervention did they occur in? To synthesise the data, juxtaposition, reconciling, adjudication, consolidation, and locating processes will be used [41,42]. Eliciting, modifying, and testing CMO settings enables a deeper and more thorough insight into why and under what conditions the programme is effective. This will provide a refined realist programme theory [23,27]. All authors will analyse the first two transcripts to build a shared understanding of what comprises a context, mechanism, and outcome in a quality and safety monitoring system. When the interpretation becomes difficult, the authors will review the study to decide, and stakeholder group discussions will help to refine the “programme theory.”

### Documentation and future research

The report will be summarised and organised to fulfill the needs of knowledge users in a final report.

### IPTs testing

The transferability of the findings across settings is one of the strengths of realist methodology. Programme theories enhance our understanding of how interventions that share similar characteristics work. Even though context-specific details can change, applying programme theories to assess similar interventions can be used as a starting point [43]. The program theories developed in this review will serve as a foundation for the design and implementation of a national quality and patient safety surveillance system. The theories will undergo further refinement through realist interviews with the project team to provide the context and allow adaptation of the programme theories for the Irish healthcare setting. The findings from the RRR will be refined/refused/confirmed with the qualitative interviews to assess the contexts to determine supportive conditions or necessary intervention points for developing successful outcomes, to inform the implementation design, or to modify specific contexts and the improvement of the QSSS programme.

A realist review is a type of systematic literature review characterised by an explanatory focus rather than a judgmental focus on the effectiveness of an intervention. A realist review is concerned with answering questions about how an intervention works, for whom it works, and under what conditions, rather than judging the intervention’s effects [27]. Pawson stated the success of the intervention; ‘For realists, the success and failure of a social programme lies in the complex relationship that exists between the resources the intervention has to offer, the context in which it was implemented, and the reactions of users.’ [44]. And they suggest stakeholder involvement in this validating process [24]. Thus, acting on the number of IPTs, a ranking exercise will be undertaken to enable the prioritisation of developed IPTs for testing. The expert panel will score the IPTs in terms of their importance. According to Pawson, workplace interventions are ongoing, active programmes that adapt to changing conditions and dynamic processes. Exploring ‘quality and safety surveillance system interventions’ in all different workstream contexts (Data Governance, System Design, Clinical Advisory Group, Change Management, and Signal Design) will allow for a broader array of factors to provide more rigorous testing of the IPTs. To evaluate the IPTs both the theory-gleaning and refinement techniques of the realist evaluation will be used in the testing phase. The program theories will be falsified or refined by allowing the interviewee’s expertise as a tool to provide a well-informed and critical account of them [45]. To prevent possible challenges in the rigor of the interview technique, the researchers will follow the following precautions; To reduce the likelihood of simply agree the researcher’s theory, the interviewees will ask to provide examples to enact a conceptual focusing function [46]; In terms of the trustworthiness of the realist interview technique, both descriptive and theoretical validity will be considered. To do that, IPTs will be developed with the experts from the programme, and the realist interviews will be verified with grey literature and meetings [47]; Finally, to determine the validity of a truth judgment, researchers will apply the concept of judgmental reality during discussions with program stakeholders [48]. Data analysis and synthesis will be informed by Gilmore et al.’s (2019) guidelines for realist evaluation [49]. Besides, RAMASES II quality and reporting standards for realist evaluation will be followed [50].

## Dissemination

The developed programme theories will be used by National policymakers and will guide future system development. Results will be disseminated by collaborating with knowledge users to put the findings into practice through policy suggestions, more research and knowledge synthesis, or knowledge application evaluation. Programme theories will also aid in describing unintended effects driven by context alterations and their subsequent interactions with mechanisms [20]. The findings will also be published in high-quality journals and presented at patient safety conferences.
